# Obstructive sleep apnoea is associated with progression of arterial stiffness independent of obesity in participants without hypertension: A KoGES Prospective Cohort Study

**DOI:** 10.1038/s41598-018-26587-y

**Published:** 2018-05-25

**Authors:** Jinkwan Kim, Seung Ku Lee, Dae Wui Yoon, Chol Shin

**Affiliations:** 1 Department of Biomedical Laboratory Science, College of Health Science, Jungwon University, Geo-San, Republic of Korea; 2 Department of Pulmonary Sleep and Critical Care Medicine Disorder Center, College of Medicine, Korea University, Ansan, Republic of Korea; 3 Institute of Human Genomic Study, Korea University Ansan Hospital, Korea University, Ansan, Republic of Korea

## Abstract

Accumulating evidence shows that obstructive sleep apnoea (OSA) is associated with an increased risk of cardiovascular disease. However, there are no published prospective studies on the relationship between OSA and the progression of arterial stiffness. We hypothesised that OSA would increase the risk of arterial stiffness progression, independent of obesity. In the present large cohort study, 1921 participants were randomly selected and underwent polysomnography. The brachial ankle pulse wave velocity (baPWV) was measured at baseline and during the follow-period using a standard protocol. Elevated baPWV was defined as a value greater than the cut-off of highest tertile level in the complete study cohort. The percentage of elevated baPWV and the ΔbaPWV significantly increased with OSA severity. After adjusting for potential confounding factors, participants with moderate-to-severe OSA without hypertension had a significantly higher risk of elevated ΔbaPWV than those without OSA. More importantly, using multivariate mixed-effect models, we found that the ΔbaPWV over 6 years significantly differed according to OSA severity. Therefore, moderate-to-severe OSA in participants without hypertension was a predictor of future burden of arterial stiffness progression, independent of obesity, suggesting that it may contribute to the increased risk of cardiovascular disease.

## Introduction

Obstructive sleep apnoea (OSA) is characterised by repeated events of partial or complete upper airway obstruction during sleep, leading to hypoxemia and sleep fragmentation. Accumulating evidence strongly supports that OSA is associated with an increased risk of cardiovascular morbidities and mortalities^1–3^. Although the exact mechanisms underlying the associations between OSA and cardiovascular disease (CVD) are still unclear, multiple factors such as increased sympathetic activity, systemic inflammation, and accumulation of reactive oxygen species (ROS) related to hypoxia-reoxygenation are mechanistically involved in the pathogenesis of CVD in OSA^4,5^.

During the past two decades, an increasing body of evidence has shown that arterial stiffness is associated with several cardiovascular risk factors including age, hypertension, diabetes mellitus (DM), and obesity. It is recognised as an early independent predictor of cardiovascular events and all-cause mortality^6,7^. Brachial ankle pulse wave velocity (baPWV), which is considered a surrogate marker of CVD, has been increasingly used in clinical as well as population-based studies owing to its simplicity and ease of measurement^8^. Unsurprisingly, substantial data from clinical or community-based studies demonstrated that pulse wave velocity (PWV) in patients with OSA is significantly high, but decreases after continuous positive airway pressure (CPAP) treatment^9–11^. Moreover, recent systemic reviews also revealed a significant association between OSA and PWV, suggesting that increased PWV might be an independent predictor of OSA-related cardiovascular complications^10,12^. However, not all studies have confirmed this putative association owing to the coexistence of various cofounding factors, such as obesity and hypertension in OSA^12,13^. Further, there has been no large prospective study investigating the association between OSA and the increased risk of arterial stiffness progression. Given the high prevalence of hypertension and obesity in patients with OSA, it is crucial that studies control for the confounding effects of these important predictors when examining a causal relationship between OSA and progression of arterial stiffness in a prospective setting. Moreover, the important pathophysiological role of arterial stiffness in CVD and modifiable determinants of OSA in progression of arterial stiffness need to be better understood by studying longitudinal changes. Therefore, we hypothesised that OSA would increase the risk of arterial stiffness progression independently of obesity in a large cohort study.

## Results

### Study population

We performed a prospective cohort study of 1,921 participants from the Korean Genome and Epidemiology Study (KoGES), which started in 2001. The participants underwent baseline polysomnography (PSG) and continued to participate for an average of 5.88 years. Of the 1,921 participants who underwent baseline examination, 258 (141 men and 117 women) dropped out over the 6-year follow-up period (non-response rate, 13.4%). There were no significant differences between respondents and non-respondents regarding smoking status, alcohol use, and body mass index (BMI; P > 0.05). However, the mean age and apnoea-hypopnea index (AHI) was a little higher in the non-respondents than in the respondents. At baseline, the participant’s mean age was 54.4 years old, and 51.0% of the participants were men. Table 1 outlines the general characteristics of the participants of the present study. The participants were divided into three groups according to severity of OSA. PSG data, including AHI and oxygen saturation (SaO_2_) nadir, showed a significant group difference (P < 0.01). Metabolic profiles, including those for glucose, high-density lipoprotein (HDL) cholesterol, and triglyceride levels, significantly differed among the three groups (P < 0.01); however, total cholesterol did not vary significantly. In addition, the percentage of hypertension and diabetes mellitus significantly increased with severity of OSA.

**Table 1:** General characteristics of study participants according to severity of obstructive sleep apnoea (OSA)^a)^.

	Non-OSA	Mild OSA	Moderate-to-severe OSA	p-value
	(AHI < 5)	(5 ≤ AHI < 15)	(AHI ≥ 15)	
Sample size, n (%)	1017 (52.9)	636 (33.1)	268 (14.0)	—
Age (years)	53.0 ± 6.5	55.8 ± 7.4^†^	56.2 ± 7.5^‡^	<0.01
Male, n (%)	424 (41.7)	366 (57.5)	190 (70.9)	<0.001
BMI (kg/m^2^)	23.8 ± 2.6	25.0 ± 2.7^†^	26.0 ± 3.1^‡,§^	<0.01
ΔBMI (kg/m^2^)*	−0.02 ± 1.1	0.01 ± 1.2	−0.15 ± 1.2	0.22
WHR (cm/cm)	0.84 ± 0.06	0.87 ± 0.06^†^	0.90 ± 0.05^‡,§^	<0.01
ΔWHR (cm)*	0.039 ± 0.04	0.038 ± 0.04	0.032 ± 0.03	0.054
FM/Body weight (kg/kg)	0.26 ± 0.6	0.27 ± 0.06	0.27 ± 0.06	0.33
ΔFM/Body weight (kg/kg)*	0.02 ± 0.1	0.01 ± 0.03	0.01 ± 0.03	0.69
ESS	5.9 ± 4.3	5.7 ± 4.4	6.0 ± 3.8	0.44
Current Smoker, n (%)	134 (13.2)	98 (15.4)	49 (18.3)	<0.01
Current Drinker, n (%)	510 (50.1)	262 (41.2)	83 (31.0)	<0.01
Hypertension, n (%)	202 (19.9)	192 (30.2)	120 (44.8)	<0.01
Diabetes Mellitus, n (%)	71 (7.0)	73 (11.5)	50 (18.7)	<0.01
Systolic BP at baseline (mmHg)	109.0 ± 13.9	112.7 ± 14.0^†^	116.0 ± 13.9^‡,§^	<0.01
Diastolic BP at baseline (mmHg)	73.4 ± 9.7	75.5 ± 9.7^†^	78.0 ± 9.4^‡,§^	<0.01
Systolic BP at follow-up (mmHg)*	113.1 ± 13.4	116.8 ± 13.4^†^	118.5 ± 13.8^‡,§^	<0.01
Diastolic BP at follow-up (mmHg)*	73.3 ± 9.0	74.6 ± 9.3^†^	76.1 ± 10.3^‡,§^	<0.01
AHI (events/hour)	1.96 ± 1.4	8.6 ± 2.7^†^	25.9 ± 11.3^‡,§^	<0.01
(Median, IQR)	(1.70, 0.7–3.1)	(8.30, 6.2–10.7)	(22.6, 17.6–29.9)	
SaO2 Nadir (%)	89.9 ± 4.6	85.4 ± 4.0^†^	81.1 ± 5.5^‡,§^	<0.01
(Median, IQR)	(91.0, 89.0–92.0)	(86.0, 83.0–88.0)	(82.0, 79.0–85.0)	
Oxygen saturation <90%	1.0 ± 8.9	2.6 ± 5.4^†^	15.8 ± 16.0^‡,§^	<0.01
Fasting Glucose (mg/dl)	96.5 ± 28.7	100.7 ± 29.3^†^	110.0 ± 42.2^‡,§^	<0.01
Total Cholesterol (mg/dl)	200.4 ± 34.0	202.1 ± 34.1	201.7 ± 35.0	0.325
HDL Cholesterol (mg/dl)	46.1 ± 10.6	44.5 ± 10.3^&^	42.6 ± 9.4^‡,§^	<0.01
Triglyceride (mg/dl)	127.4 ± 76.9	148.9 ± 93.1^†^	167.6 ± 97.0^‡,§^	<0.01
HbA1c (%)	5.56 ± 0.70	5.73 ± 0.81^†^	5.93 ± 0.89^‡,§^	<0.01

Abbreviation: BMI, body mass index; WHR, waist to hip ratio; FM, fat mass; ESS, Epworth sleepiness scale; BP, blood pressure; AHI, apnoea hypopnea index; IQR, interquartile range; HDL, high-density lipoprotein.

^a)^Scale variables are expresses as mean ± SD; Statistical significance was estimated after log transformation.

*A total of 1663 participants were included in the analysis.

^†^P < 0.01, Mild OSA vs. Non-OSA.

^‡^P < 0.01, Moderate-to-severe OSA vs. Non-OSA.

^§^P < 0.01, Moderate-to^-^severe OSA vs. Mild OSA.

^&^P < 0.05, Mild OSA vs. Non-OSA.

### Odds ratios for the risk of elevated baPWV according to OSA severity in the presence or absence of hypertension at baseline

Table 2 shows the baPWV data for the three groups, divided by the severity of OSA, in the presence or absence of hypertension at baseline. Mean baPWV significantly increased with OSA severity in participants without hypertension (non-OSA vs. mild OSA vs. moderate-to-severe OSA, 13.0 ± 1.7 vs. 13.7 ± 1.9 vs. 13.9 ± 1.8 m/s, respectively, P < 0.01), but not in those with hypertension. Elevated baPWV was defined as a baPWV value greater than the cut-off of highest tertile level in the whole cohort of participants. The percentage of elevated baPWV significantly differed in a dose-dependent manner according to OSA severity, in the group without hypertension (non-OSA vs. mild OSA vs. moderate-to-severe OSA, 19.6% vs. 33.8% vs. 44.6%, respectively, P < 0.01). Univariate and multiple logistic regression analyses were performed to estimate the odds ratios for the likelihood of elevated baPWV by OSA severity in the presence or absence of hypertension. From the multivariate analyses, after adjusting for age, sex, smoking, alcohol status, mean arterial pressure, fasting glucose, HDL cholesterol, DM medication, and each of the obesity-related variables (BMI, waist-to-hip ratio [WHR], and FM/body weight), we found that participants with moderate-to-severe OSA (AHI ≥ 15) in the group without hypertension had a 2.60-(BMI adjusted, 95% CI, 1.66–4.05, P < 0.01), 2.20-(WHI adjusted, 95% CI, 1.42–3.39, P < 0.01), and 2.38-(FM/body weight adjusted, 95% CI, 1.53–3.68, P < 0.01) fold increased risk for elevated baPWV, compared to those without OSA (AHI < 5).

**Table 2:** Mean brachial ankle pulse wave velocity (baPWV) and odds ratios for the risk of elevated baPWV according to severity of obstructive sleep apnoea (OSA) in participants with or without hypertension at baseline.

		Odds ratios for the risk of elevated baPWV (95% CI)^a)^		
		Non-OSA	Mild OSA	Moderate-to-severe OSA
		(n = 1017)	(n = 636)	(n = 268)
With Hypertension (n = 514)	Sample size, n (%)	202 (39.3)	192 (37.4)	120 (23.3)
	baPWV (m/s)	14.9 ± 2.4	14.6 ± 2.1	14.8 ± 2.1
	Elevated baPWV n, (%)^a)^	123 (56.2)	104 (54.2)	67 (55.8)
	Unadjusted	Reference	0.92 (0.62–1.36)	0.98 (0.63–1.54)
	Model 1	Reference	0.80 (0.50–1.27)	0.76 (0.44–1.31)
	Model 2	Reference	0.70 (0.44–1.11)	0.63 (0.37–1.07)
	Model 3	Reference	0.76 (0.48–1.20)	0.70 (0.41–1.20)
Without Hypertension (n = 1407)	Sample size, n (%)	815 (57.9)	444 (31.6)	148 (10.5)
	baPWV (m/s)^†^	13.0 ± 1.7	13.7 ± 1.9	13.9 ± 1.8
	Elevated baPWV n, (%) ^a),†^	170 (19.6)	150 (33.8)	66 (44.6)
	Unadjusted	Reference	2.08 (1.61–2.70)^&^	3.29 (2.28–4.74)^&^
	Model 1	Reference	1.53 (1.12–2.09)^&^	2.60 (1.66–4.05)^&^
	Model 2	Reference	1.39 (1.02–1.89)^§^	2.20 (1.42–3.39)^&^
	Model 3	Reference	1.45 (1.06–1.98)^§^	2.38 (1.53–3.68)^&^

^&^P < 0.01, ^§^P < 0.05, ^†^Significant difference between groups (P < 0.01).

^a)^Elevated baPWV defined as a value greater than the cut-off of the highest tertile level.

Model 1: Adjusted for age, sex, smoking, alcohol status, mean arterial pressure, fasting glucose, HDL cholesterol, DM medication, and BMI.

Model 2: Adjusted for age, sex, smoking, alcohol status, mean arterial pressure, fasting glucose, HDL cholesterol, DM medication, and WHR.

Model 3: Adjusted for age, sex, smoking, alcohol status, mean arterial pressure, fasting glucose, HDL cholesterol, DM medication, and FM/body weight.

### Odds ratios for the risk of elevated ΔbaPWV according to OSA severity in the presence or absence of hypertension at the 6-year follow-up

Table 3 outlines the mean baPWV and ΔbaPWV at the 6-year follow-up across OSA severity. There was a significant difference among groups in the mean baPWV and ΔbaPWV at the 6-year follow-up in participants with hypertension (non-OSA vs. mild OSA vs. moderate-to-severe OSA, 13.8 ± 2.0 vs. 14.6 ± 2.3 vs. 15.4 ± 2.7 m/s for baPWV, P < 0.01, 0.81 ± 1.4 vs. 0.85 ± 1.6 vs. 1.55 ± 2.0 m/s for ΔbaPWV, P < 0.01). Multivariate analysis with adjustment for age, sex, smoking, alcohol status, mean arterial pressure, fasting glucose, HDL cholesterol, DM medication, and each of obesity-related variables (BMI, WHR, and FM/body weight) and their changes revealed that the group with moderate-to-severe OSA had a 2.12-(BMI and ΔBMI adjusted, 95% CI, 1.39–3.23, P < 0.01), 2.00-(WHI and ΔWHI adjusted, 95% CI, 1.31–3.02, P < 0.01), and 2.03-(FM/body weight and ΔFM/body weight adjusted, 95% CI, 1.34–3.08, P < 0.01) fold increase in the risk of elevated ΔbaPWV, compared to non-OSA group participants without hypertension.

**Table 3:** Mean ΔbaPWV and odds ratios for the risk of elevated ΔbaPWV according to severity of OSA in participants with or without hypertension at the 6-year follow-up.

		Odds ratios for the risk of elevated ΔbaPWV (95% CI)^a)^		
		Non-OSA	Mild OSA	Moderate-to-severe OSA
		(n = 906)	(n = 536)	(n = 221)
With Hypertension (n = 421)	Sample size, n (%)	170 (40.4)	156 (37.1)	95 (22.6)
	baPWV (m/s)	15.6 ± 2.7	15.6 ± 2.4	15.8 ± 3.3
	ΔbaPWV (m/s)	0.73 ± 2.4	1.07 ± 2.1	1.13 ± 2.5
	Elevated ΔbaPWV n, (%)^a)^	55 (32.4)	68 (43.6)	31 (32.6)
	Unadjusted	Reference	1.61 (1.02–2.53)^§^	1.01 (0.59–1.73)
	Model 1	Reference	1.52 (0.97–2.34)	1.00 (0.54–1.82)
	Model 2	Reference	1.55 (0.95–2.53)	0.93 (0.51–1.67)
	Model 3	Reference	1.53 (0.93–2.51)	0.90 (0.49–1.65)
Without Hypertension (n = 1242)	Sample size, n (%)	736 (59.3)	380 (30.6)	126 (10.1)
	baPWV (m/s)^†^	13.8 ± 2.0	14.6 ± 2.3	15.4 ± 2.7
	ΔbaPWV (m/s)^†^	0.81 ± 1.4	0.85 ± 1.6	1.55 ± 2.0
	Elevated ΔbaPWV n, (%)^a),†^	214 (29.1)	116 (30.5)	61 (48.4)
	Unadjusted	Reference	1.07 (0.81–1.40)	2.28 (1.55–3.36)^&^
	Model 1	Reference	0.93 (0.70–1.25)	2.12 (1.39–3.23)^&^
	Model 2	Reference	0.91 (0.68–1.21)	2.00 (1.31–3.02)^&^
	Model 3	Reference	0.92 (0.69–1.24)	2.03 (1.34–3.08)^&^

P < 0.05, ^&^P < 0.01, ^†^Significant difference between groups (P < 0.01).

^a)^Elevated ΔbaPWV defined as greater than the cut-off of highest tertile level of ΔbaPWV.

Model 1: Adjusted for age, sex, smoking, alcohol status, mean arterial pressure, BMI, fasting glucose, HDL cholesterol, DM medication at baseline and change in BMI (ΔBMI) at the 6-year follow-up.

Model 2: Adjusted for age, sex, smoking, alcohol status, mean arterial pressure, WHR, fasting glucose, HDL cholesterol, DM medication at baseline and change in WHR (ΔWHR) at the 6-year follow-up.

Model 3: Adjusted for age, sex, smoking, alcohol status, mean arterial pressure, FM/body weight, fasting glucose, HDL cholesterol, DM medication at baseline and change in FM/body weight (ΔFM/body weight) at the 6-year follow-up.

### Change in baPWV according to OSA severity across the time estimated by the mixed effects model

**Figure 1:**
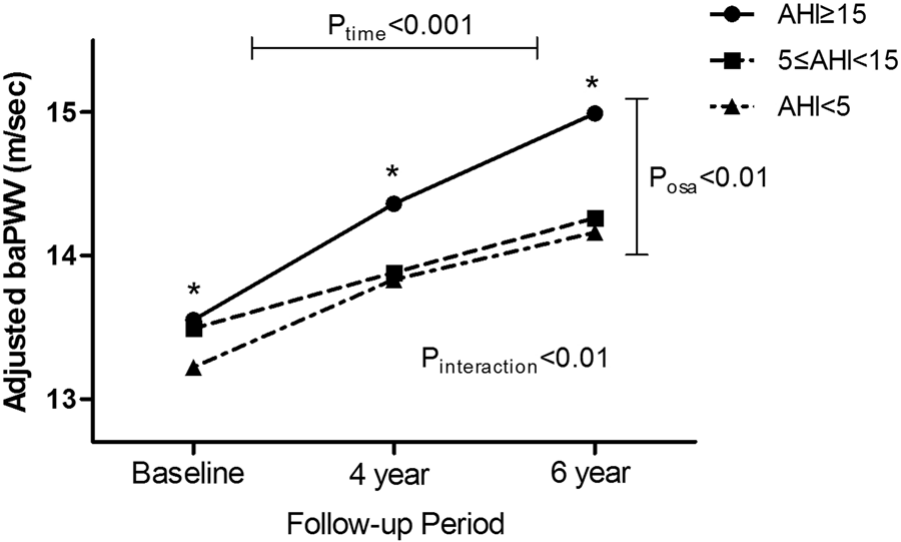
Change in mean brachial ankle pulse wave velocity (baPWV) over the 6-year follow-up period according to severity of obstructive sleep apnoea (OSA) in participants without hypertension (n = 1242). P-values were derived from multivariate mixed-effect linear regression models adjusting for age, sex, smoking, alcohol status, mean arterial pressure, body mass index (BMI), fasting glucose, high-density lipoprotein (HDL) cholesterol, diabetes mellitus (DM) medication at baseline and change in BMI at the 6-year follow-up. P_time_ and P_osa_ indicate the effect of time on change in baPWV over 6 years and the effect of OSA groups on baPWV across time, respectively; P_interaction_ indicates whether the change in baPWV over time differs significantly among OSA groups. *Adjusted mean baPWV in the moderate-to-severe OSA group is significantly different from that in the non-OSA group, according to pairwise comparisons.

Figure 1 presents the change in adjusted mean baPWV over 6 years among three groups stratified by OSA severity, in participants without hypertension, adjusting for age, sex, smoking, alcohol status, mean arterial pressure, BMI, fasting glucose, HDL cholesterol, DM medication at baseline, and change in BMI at the 6-year follow-up. Interestingly, the P-value, derived from mixed effects linear regression analyses indicated that adjusted mean baPWV increased with time (P_time_ < 0.01). Further, groups with OSA showed elevated baPWV across time (P_osa_ < 0.01). More importantly, the pattern of change in the adjusted mean baPWV over the 6 years significantly differed according to severity of OSA (P_interaction_ < 0.01). Pairwise comparisons revealed that the adjusted mean baPWV in participants with moderate-to-severe OSA was significantly higher than those in non-OSA at 4 and 6 years, as well as at baseline.

## Discussion

In a large prospective cohort study, we found that moderate-to-severe OSA was not only closely associated with elevated baPWV, but also significantly related to elevated ΔbaPWV in participants without hypertension over a 6-year follow-up period, independent of obesity. Interestingly, a linear mixed model estimating the mean ΔbaPWV over time based on OSA severity in participants without hypertension, with adjustment for age, sex, smoking, alcohol status, mean arterial pressure, BMI, fasting glucose, HDL cholesterol, DM medication at baseline and change in BMI at the 6-year follow-up, showed that the pattern of change in the adjusted mean baPWV over time significantly differed according to OSA severity (Fig. 1). In pairwise comparisons, adjusted mean baPWV in patients with moderate-to-severe OSA was significantly higher than that in those without OSA at 4 and 6 years, as well as at baseline, suggesting that moderate-to-severe OSA is an important predictor for accelerating the progression of arterial stiffness independent of obesity in participants without hypertension. To the best of our knowledge, this is the first study to investigate the association between OSA severity and progression of arterial stiffness in a large prospective study.

Over several decades, arterial stiffness has been increasingly recognised as an important prognostic index and potential therapeutic target for CVD. The elastic properties of arteries, which are dependent on the stiffness of the arterial walls, allow for the smoothing of oscillations in blood pressure, reduction of the pulse pressure, and perfusion of the myocardium^8,14–16^. Although arterial stiffness can be evaluated using various parameters, there is currently no consistent method due to complexities in accurately measuring the stress and strain in arteries. Among these methods, PWV has been widely considered as an index of arterial stiffness owing to its simplicity, reproducibility, and inexpensiveness^17,18^. A larger number of previous studies suggested that increased aortic PWV has been shown to predict cardiovascular morbidity and mortality in individuals with end stage renal failure, hypertension, and diabetes mellitus in the general population^14,16^. Not surprisingly, a majority of substantial evidence demonstrated a significant association between OSA severity and arterial stiffness in clinical and community-based studies^11,12,19,20^. However, the findings are not clearly established owing to lack of sufficient sample size, cross-sectional design, and inadequate controlling of confounders, such as hypertension and obesity. Some studies with a case-control setting failed to establish a significant difference in arterial stiffness between groups with OSA severity after adjusting for various covariates^12,13^. Moreover, no prospective studies have investigated a causal relationship by demonstrating an accelerated progression of arterial stiffness using PWV among participants with OSA. Considering the increasing body of evidence demonstrating that OSA, hypertension, and obesity share many common pathophysiological pathways inflicting damage to the cardiovascular system^21^, it is crucial that studies attempt to control for the confounding effects in progression of arterial stiffness according to OSA severity^22^. In this context, several studies have also examined the association between OSA and arterial stiffness independently from various confounding effects, such as hypertension and obesity, by excluding patients with these conditions^10,22–24^. In the present study, to examine whether OSA is responsible for elevation of arterial stiffness independent of obesity, we separately tested three obesity-related variables (BMI, WHR, and FM/body weight) in regression models. In public health and population-based studies these variables are commonly useful indices of obesity and central obesity. As a result, we observed that moderate-to-severe OSA is closely related to increased risk of arterial stiffness, which is independent of obesity, in participants without hypertension, both at baseline and at the 6-year follow-up, suggesting that moderate-to-severe OSA may potentially contribute to an exacerbated risk of CVD. However, we did not find significant association between OSA severity and progression of arterial stiffness in the group with hypertension. Thus, we postulated that anti-hypertensive medication might affect the progression of arterial stiffness in OSA because our data showed that the percentage of participants taking any type of anti-hypertensive medication was highest (non-OSA vs. mild OSA vs. moderate-to-severe OSA, 69.8% vs. 79.2% vs. 85.0%, P < 0.01) in the hypertensive group with moderate-to-severe OSA. Previous studies demonstrated that medications lowering blood pressure were likely to favourably influence arterial stiffness through the passive decrease in applied distending stress^10^. However, the magnitude of the arterial stiffness effects for hypertension medication is not always equivalent. No studies have attempted to show which antihypertensive medications work best in reducing blood pressure and arterial stiffness in OSA^11^. Thus, additional studies are required to further explore the magnitude and significance of anti-hypertensive medication on the progression of arterial stiffness or development of CVD according to OSA severity in a large prospective study.

Although the pathophysiological mechanisms by which OSA leads to accelerate progression of arterial stiffness are not clearly evident, the activation of inflammatory pathways and reactive oxygen species (ROS) have been proposed to play critical roles^10,25^. During the past two decades, substantial evidence has suggested that the intermittent hypoxia and reoxygenation that characterize OSA contribute to the cumulative burden of oxidative stress and generation of ROS, which are of particular importance due to their potency and ability to regulate small and large artery stiffness^25^. Moreover, the deterioration of endothelial function due to increased inflammation by OSA is associated with a decrease in endothelial nitric oxide bioavailability, which contributes to impaired endothelial-dependent vasoreactivity^10,11^. It is also reported that various inflammatory markers, such as C-reactive protein (CRP), TNF-α, and IL-6 are associated with arterial stiffness indices, including aortic PWV and augmentation index^11^. Therefore, the combined effect of OSA and inflammatory or other makers for oxidative stress on increased risk of arterial stiffness should be performed in future prospective studies.

The present study has several strengths that lend confidence to the findings. This study was a large prospective cohort study, which allowed us to investigate a causal relationship between OSA severity and progression of arterial stiffness in general population. In addition, we selected 1,921 participants from the KoGES cohort, and not from sleep clinics or a community-based population, making it possible to obtain results that were more representative of the general population. Further, we used the same baPWV measurement and evaluated the adiposity by standard protocol at baseline and 6-year follow-up. Another advantage of the present study was that the evaluation of OSA was performed using a portable PSG at home, which provided a more realistic evaluation of OSA severity than hospital-based studies, owing to the maintenance of regular daily habits of sleep, physical activity, and diet in the general population.

Although the present study included a large general population sample, prospective design, and follow-up measurements, several limitations should also be acknowledged. First, the evaluation of arterial stiffness using baPWV among those with OSA may not sufficiently represent the arterial stiffness of central artery because of its anatomical properties, including long muscular artery. Further, although the carotid–femoral PWV is considered the golden standard and widely used for measuring the stiffness of central arteries and predictor for cardiovascular events, recent studies suggested that baPWV exhibits a high correlation with cfPWV and similarly has a strong ability to predict future cardiovascular events^8,15,26^. This suggests that it may be more easily applied in clinical or population based studies than cfPWV owing to the advantages of simplicity and ease of its measurement^8^. Second, we did not exclude the possible effect of circadian variation on arterial stiffness. Several studies have demonstrated that arterial stiffness is higher in the morning as opposed to the evening in patients with OSA, suggesting that circadian effect may represent a carryover effect from increased stiffness as well as blood pressure observed during respiratory disturbances at night^11,27^. However, the lack of circadian variation of PWV measurements in young healthy volunteers has also been reported^28^. Thus, additional studies on the influence of circadian variations for PWV measurement are required. Third, the elevation of baPWV in the general population may be influenced in part by a participant’s exercise status; these factors were not considered in the present study. Several lines of research suggest that aerobic exercise significantly improved arterial stiffness. Further, this effect seems to be enhanced with higher aerobic exercise intensity and in participants with greater arterial stiffness^29^. Accordingly, future longitudinal studies focussing on interactions between arterial stiffness and physical activity in participants with OSA should be conducted. Finally, we did not investigate the possibility of reverse causality, i.e., whether continuous positive airway pressure (CPAP) treatment for OSA could reduce or delay the progression of arterial stiffness, thereby reducing the risk of any OSA-related comorbidities. Although a number of studies have shown that CPAP treatment is associated with a decrease in arterial stiffness using various techniques, several studies have produced conflicting results, owing to methodological limitations, such as small sample sizes, OSA severity, variance in treatment duration, or inadequate consideration of various confounding factors^9,11^. Thus, further additional randomised intervention studies with a larger sample size and sufficient duration of CPAP treatment are needed to address whether CPAP treatment is significantly associated with the decreased progression of arterial stiffness and to evaluate if a significant reduction in later cardiovascular events may be predicted early in treatment by measures of arterial stiffness.

Taken together, individuals with moderate-to-severe OSA without hypertension had a higher risk of arterial stiffness progression, independent of obesity. An additional longitudinal study is required to further address the significance and magnitude of OSA on accelerating arterial stiffness in the context of reducing CVD risk.

## Methods

### Participants

The Korean Genome and Epidemiology Study (KoGES), an ongoing prospective cohort study, was initiated in 2001 to examine the risk and burden of chronic disease among the general Korean population. Detailed information on the study design and aims of the KoGES has been previously reported^30–33^. In brief, participants of the current study included individuals who enrolled in the Ansan cohort, which consists of residents of a suburban community of Ansan City, which is 32 km southwest of Seoul. From June 2001 to January 2003, a longitudinal cohort was formed, consisting of 5,015 participants who participated in a comprehensive health examination and on-site interviews at Korea University Ansan Hospital. Follow-up examinations were performed biennially with scheduled site visits. At each visit, participants signed an informed consent form. This study was approved by the Korea University Ansan Hospital Human Research Committee (Protocol ID: ED0624). All methods and experiments were performed in accordance with the relevant guidelines and regulations. In the present study, data from the 4^th^ biennial examination from March 2007 to February 2009 and the 7^th^ examination from March 2013 to February 2015 (follow-up) were used. Participants in the present study included only those with PSG data acquired between September 2009 and February 2012. After excluding participants who had missing data and those with extreme outliers of biochemical data, a total of 1,921 individuals (980 men and 941 women) were recruited into the current study. Participants with a known defined systemic inflammatory disease or genetic abnormality, or those who received any treatment for OSA were excluded. Participants were divided into three groups according to the severity of OSA for the present study.

### Overnight polysomnography

An overnight PSG was performed at each participant’s home using a portable device (Embletta® X-100; Embla Systems, San Carlos, CA, USA) as previously described^31,33^. The recording channels were as follows: one electroencephalography (C4-A1), one electrooculography, one chin electromyography, one modified lead II electrocardiography, one airflow from nasal airflow pressure transducer, two respiratory chest and abdominal respiratory inductance plethysmography, one pulse oximeter, and one position sensor. Obstructive apnoea was defined as a clear decrease (≥90%) from baseline in the amplitude of the nasal pressure with ongoing chest and abdominal movement. Hypopnea was identified as a reduction of ≥30% in the nasal pressure from baseline, associated with at least 4% oxygen desaturation on pulse oximetry. The duration threshold for these respiratory events was 10 s. OSA was defined if the AHI score was greater than 5; mild OSA and moderate-to-severe OSA were defined as AHI >5 and ≥15, respectively. Arousals were scored according to the American Academy of Sleep Medicine Scoring Manual^34,35^.

### Anthropometric, biochemical data, and assessment of body composition

All study participants provided personal health history, sleep habits, and lifestyle information. Daytime sleepiness was assessed with the Epworth Sleepiness Scale (ESS). Blood was drawn for biochemical analysis after overnight fasting. Plasma glucose, serum triglycerides, and HDL cholesterol were measured with an auto-analyser (ADVIA 1650 and 1800, Siemens, Tarrytown, NY). BMI and WHR were calculated as the weight divided by the square of height and as waist divided by hip. Body composition was also measured by means of multi-frequency bioelectrical impedance analysis (BIA) with 8-point tactile electrodes (InBody 720; Biospace, Seoul, Korea)^31,36,37^. The analyser used an alternating current of 250 mA at variable frequencies of 1, 5, 50, 250, 500, and 1,000 kHz. Fat-free mass and fat mass were obtained from a multi-frequency BIA.

### Subclinical measurement of arterial stiffness

At baseline and biennial follow-up visits, blood pressure (BP) was measured by trained examiners according to a standardised protocol following a rest period of at least 5 min in the sitting position using an appropriate-sized cuff and mercury sphygmomanometer^30^. Hypertension was defined when systolic blood pressure was 140 mmHg (or higher) and diastolic blood pressure was 90 mmHg (or higher), or with antihypertensive medication use. DM was also defined when fasting glucose level was higher than 126 mg/dl or with anti-hyperglycaemic medication use. Participants with hypertension were allowed to take anti-hypertensive medication as prescribed by their physician. BaPWV, which was used to assess arterial stiffness, was measured using a standardised protocol at baseline and repeated at follow-up (i.e., at 4 and 6 years). A non-invasive automated waveform analyser (VP1000; Omron Co., Japan) was used with appropriate-size cuffs. After 10 min of rest in the supine position, occlusion and monitoring cuffs were placed around both the arms and ankles of the participants. The baPWV was determined as the distance between arterial sites divided by the time between the feet of the respective waveforms^38,39^. Elevated baPWV was defined as greater than the cut-off of highest tertile level (>14.2 m/s) of the whole study cohort. Moreover, ΔbaPWV was defined as a change in baPWV at the 6-year follow-up; elevated ΔbaPWV also defined as higher than the cut-off of highest tertile level of ΔbaPWV (>1.3 m/s) in the entire participant cohort.

### Statistical analyses

Data were expressed as mean ± SD. Statistical mean difference was examined for normal variates using an analysis of variance; probabilistic distribution was compared for non-normal variates by using the Kolmogorov-Smirnov test. Multivariate logistic regression was applied, adjusting for factors identified to be significant risk factors for the elevation of baPWV or ΔbaPWV, including the following: age, sex, smoking, alcohol status, mean arterial pressure, each of obesity related variables (BMI, WHR, and fat mass), fasting glucose, HDL cholesterol, DM medication at baseline, and change in each of obesity related variables (BMI, WHR, and fat mass) at the 6-year follow-up. Adjusted odds ratios were estimated using 95% confidence intervals (CIs), with reference to that of the participants with non-OSA. Moreover, we applied linear mixed models to estimate the mean ΔbaPWV over time by OSA severity, adjusting for age, sex, smoking, alcohol status, mean arterial pressure, BMI, fasting glucose, HDL cholesterol, DM medication at baseline and change in BMI at the 6-year follow-up. P-values < 0.05 were considered indicators of statistical significance. All statistical analyses were performed using SPSS (version 23.0, IBM Corp., Armonk, NY, USA).

## Electronic supplementary material

Supplementary Figure 1. Flow chart of study participants
